# Draft Genome Sequence of *Marinobacter hydrocarbonoclasticus* Strain STW2, a Polycyclic Aromatic Hydrocarbon-Degrading and Denitrifying Bacterium from the Rhizosphere of Seagrass *Enhalus acodoides*

**DOI:** 10.1128/genomeA.01412-16

**Published:** 2017-02-23

**Authors:** Juan Ling, Liyun Lin, Yanying Zhang, Xiancheng Lin, Manzoor Ahamad, Weiguo Zhou, Junde Dong

**Affiliations:** a CAS Key Laboratory of Tropical Marine Bio-resources and Ecology (LMB), Guangdong Provincial Key Laboratory of Applied Marine Biology (LAMB), South China Sea Institute of Oceanology, Chinese Academy of Sciences, Guangzhou, China; b Tropical Marine Biological Research Station in Hainan, South China Sea Institute of Oceanology, Chinese Academy of Sciences, Sanya, China; c University of Chinese Academy of Sciences, Beijing, China

## Abstract

Here, we report the draft genome sequence of *Marinobacter hydrocarbonoclasticus* strain STW2, which was isolated from the rhizosphere of seagrass *Enhalus acodoides*. This study will facilitate future studies on the genetic pathways of marine microbes capable of both polycyclic aromatic hydrocarbon degradation and nitrate reduction.

## GENOME ANNOUNCEMENT

The species *Marinobacter hydrocarbonoclasticus*, a member of the gamma group of the proteobacteria, was first identified and described by Gauthier et al. in 1992 (1). It is a Gram-negative, rod shaped, aerobic, motile, and non-spore-forming bacterium which is widely distributed in a marine environment (1). *M. hydrocarbonoclasticus* can also grown on eicosane at high salinity (2). This strain can form bioﬁlms on hydrophobic organic compounds and possesses efficient denitrifying ability (3, 4). Due to its ability to degrade hydrocarbons, it has become a research interest in the field of marine ecology (2–4).

Analysis of the 16S rRNA gene sequence along with physiological and biochemical characteristics enabled us to identify strain STW2 as an *M. hydrocarbonoclasticus* species. Strain STW2 exhibits 97.90% similarity using BLASTn with the type strain of *M. hydrocarbonoclasticus*, ATCC 49840 (GenBank accession no. F0203363) (1, 5). In this study, *M. hydrocarbonoclasticus* strain STW2 was isolated from the rhizosphere sediment of seagrass *Enhalus acodoides* (N18°24′25.08″, E110°0′30.02″) and has been deposited in the China Center for Type Culture Collection (CCTCC) with accession number CCTCC M 2014339 (http://www.cctcc.org/index.php).

Sequences were obtained and sequenced on the Illumina HiSeq 2000 sequencing platform (Illumina, San Diego, CA, USA). Two different types of libraries were constructed for sequencing, one paired-end library (500-bp insert size) and a mate-paired library (2.5-kb insert size). Paired-end libraries were sequenced at 137× coverage (300.00 Mb clean data), and the mate-pair library was sequenced at 68× coverage (601.66 Mb clean data); the average read length was 102 bp for both libraries. SOAP*denovo* (version 2.04) was used to assemble the reads after ﬁltering (6, 7).

The ﬁnal assembly genome size of strain STW2 was 4.39 Mb in length, having 99 scaffolds and an *N*_50_ of 3,386,954 bp. The calculated G+C content of the genome was 57.04%. There were 3,939 protein-coding genes (CDSs) (958 bp average length, 89.14% coding density), 50 tRNA genes, and six rRNA genes predicted from this assembly (6–8). Further investigation on strain STW2 in combined biochemical characteristics with degrading ability of polycyclic aromatic hydrocarbons (PAHs) will help promote many beneﬁcial applications, especially the protection and restoration of the marine ecosystem.

### Accession number(s).

This whole-genome shotgun project has been deposited at DDBJ/ENA/GenBank under the accession no. MPKY00000000. The version described in this paper is version MPKY01000000.
